# Repeated transcranial magnetic stimulation on the bilateral cerebellum to improve symptoms of ataxia with multiple system atrophy: a prospective, randomized, sham-controlled pilot study

**DOI:** 10.1007/s10072-025-08001-4

**Published:** 2025-01-24

**Authors:** Dongrui Li, Changchun Jiang, Jiahui Liu, Yu Fan, Xiwa Hao, Meng Fu, Ying Xu, Xianpeng Chen, Jinfeng Zhang, Guorong Liu

**Affiliations:** 1 Baotou Medical College Center Clinical Medical College, Baotou, Inner Mongolia China; 2 Department of Neurology, Baotou Central Hospital, Baotou, Inner Mongolia China; 3 Baotou Clinical Medical College, Inner Mongolia Medical University, Baotou, Inner Mongolia China

**Keywords:** Cerebellar subtype of multiple system atrophy, Repetitive transcranial magnetic stimulation, Ataxia, Fatigue, Depression, Anxiety

## Abstract

**Objectives:**

Ataxia is a common symptom in patients with Cerebellar subtype of Multiple system atrophy (MSA-C), but effective treatments remain elusive. The present study aims to investigate whether repetitive transcranial magnetic stimulation (rTMS) over the bilateral cerebellum could relieve ataxia in patients with MSA-C.

**Patients and methods:**

This is a single-center, randomized and double-blind trial. 26 patients with MSA-C were randomly divided into experimental group and control group. The experimental group underwent (rTMS) in both cerebellum for 10 consecutive days, while the control group was given sham rTMS. The participants underwent clinical assessments at baseline (T0), and three follow-up timepoints, that is, immediately after the tenth treatment session (T1), 2 weeks (T2), and 4 weeks (T3) after T1. The Scale for the Assessment and Rating of Ataxia scores (SARA) was used as the primary outcome measure, with the Fatigue Severity Scale-9 (FSS-9), the Hamilton Anxiety Scale (HAMA) and the Hamilton Depression Rating Scale-24 (HAMD-24) as secondary outcomes.

**Results:**

Two-way repeated ANOVAs showed significant group × time interactions among SARA (p < 0.001), FSS-9 (p < 0.001), HAMA (p < 0.001) and HAMD-24 (p < 0.001). *Post-hoc* analyses showed that compared with T0, the activity group showed significant improvement in SARA, HAMA and HAMD-24 scores at T1, T2 and T3, and significant improvement in FSS-9 scores at T1 and T2, but no significant improvement in T3.

**Conclusion:**

rTMS over bilateral cerebellum could provide short-term improvements for alleviating ataxia and the symptoms of fatigue, depression anxiety, but the beneficial effects last no more than 4 weeks.

## Introduction

Multiple system atrophy (MSA) is a degenerative neural system illness that usually manifests as sporadic, slow-moving, adult-onset conditions. The prognosis is dismal, with most patients dying from complications including as severe infections, respiratory problems, and organ failure, and the average survival duration is only five to ten years[1]. The typical clinical signs of MSA include autonomic dysfunction, cerebellar ataxia, cognitive impairment, and Parkinson-like symptoms. Medication, symptomatic treatment, and neurological rehabilitation are currently the main therapeutic approaches for treating autonomic dysfunction and Parkinson's syndrome, but their efficacy is limited. There is currently no effective treatment for the symptoms experienced by patients with MSA, and to improve patient outcomes and quality of life while delaying disease progression, new and effective therapies are desperately needed for the clinical treatment process. Repetitive transcranial magnetic stimulation (rTMS) is an innovative neuroregulatory therapy that has been progressively developed and utilized over the last decade. rTMS specifically regulates neurons and their associated brain networks by using electric currents produced by electromagnetic fields. rTMS increases both short-term and long-term neuroplasticity[2], while also altering the excitability of specific areas of the cortex and the overall brain network.

Currently, extensive research has been conducted on the application of rTMS to activate the cerebral cortex for treating various conditions such as Parkinson's disease, multiple sclerosis, Alzheimer's disease, neuropathic pain, aphasia, and swallowing dysfunction resulting from stroke, as well as sleep disorders, anxiety disorders, and depression[3]. rTMS has also been utilized to stimulate the left dorsolateral prefrontal cortex (DLPFC) mitigate fatigue and motor symptoms in patients with MSA[4]. However, studies in which rTMS is used to activate cerebellar locations in MSA-C patients to mitigate symptoms of ataxia, fatigue, anxiety, and depression are lacking. Our goal was to investigate the impact of rTMS on the cerebellum to relieve motor dysfunction and other symptoms. Therefore, in this study, cerebellar dysfunction was considered the main cause of symptoms such as ataxia and motor imbalance in the case of cerebellar atrophy indicated by head MRI. We hypothesized that rTMS over the bilateral cerebellum can mitigate the symptoms of ataxia, fatigue, anxiety, and depression in MSA-C patients.

## Method

### Participants

This study encompassed 26 patients diagnosed with probable MSA-C, recruited from the Department of Neurology at Baotou Central Hospital in the Inner Mongolia Autonomous Region. Data were collected from January 2018 to May 2024. Participants were randomly assigned to either the study group (*n* = 13) or the control group (*n* = 13). The allocation of patients, clinicians, and experimenters was blinded. The Ethics Committee of Baotou Central Hospital approved and monitored this experimental study methodology. The present study was registered at the Chinese Clinical Trial Registry (https://www.chictr.org.cn/index.html, ChiCTR2400085610). The inclusion criteria were as follows: (1) the patient be classified as a probable MSA-C type according to The Movement Disorder Society Criteria for the Diagnosis of Multiple System Atrophy published in 2022[5]. (2) Age range of 50 to 75 years. (3) Absence of other neurodegenerative diseases. The exclusion criteria were as follows: (1) The presence of an unstable neurological or mental condition, either independently or in conjunction with a clinical illness. (2) Conducting a screening process to identify clinically significant abnormal test results, such as anomalies in electrocardiograms or laboratory tests. (3) A documented history of substance misuse, along with either a lack of legal capacity or limited legal competence. (4) Individuals with a history of cranial metal implants, bio-magnetic implants, metal-coated implants, neurosurgery, epilepsy, claustrophobia, or prior use of medications like bupropion or other substances that may increase the risk of TMS-induced seizures. (5) Positive pregnancy tests were observed. (6) Mini-Mental State Examination (MMSE) scores below 24 points. (7) Participation in another study concurrently with this trial. (8) Patients who had received any treatment for ataxia, anxiety, depression, or fatigue in the past six months. At the time of enrollment, some patients were taking oral medications that would not have influenced our findings. Prior to the commencement of the study, all patients provided their informed consent and data released consent. All patients were successfully completed the entire study without experiencing any adverse events during therapy.

### Experimental design

**Fig. 1:**
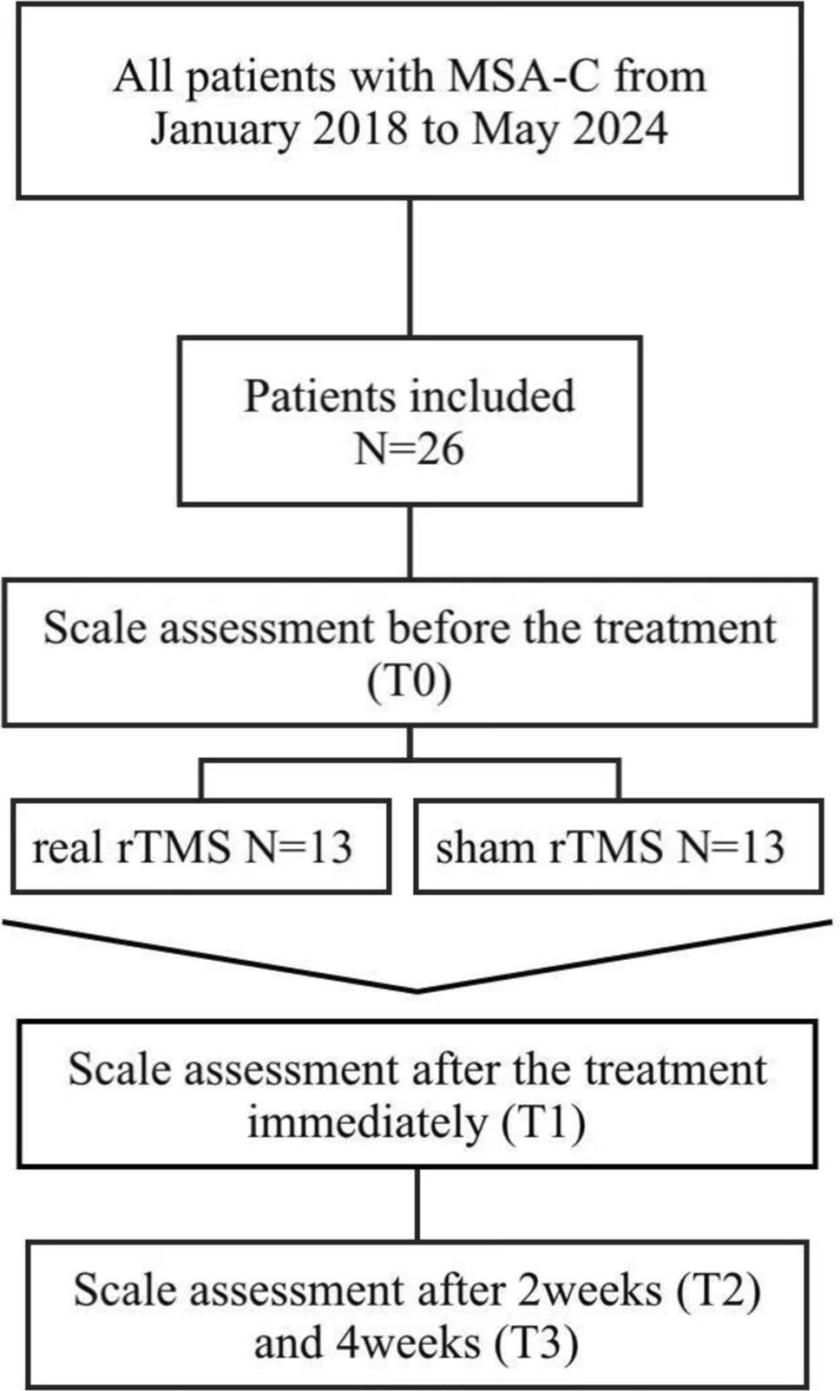
Schematic representation of experimental design

This is a single-center, randomized, double-blind experiment. A total of twenty-six patients diagnosed with MSA-C were assigned random numbers and then divided into two groups, namely the study group (n = 13) and the control group (n = 13), using a 1:1 ratio model (Fig. 1). The study group received iTBS for a duration of 10 days to activate both sides of the cerebellum, whereas the control group received sham stimulation. The treatment plan of patients was only known to rehabilitation therapist, while patients and other researchers remained blinded to both the treatment plan and the randomized sequence. Additionally, a blinded evaluation method was employed to assess the therapy's effectiveness.

### rTMS and Sham protocols

TMS was applied with a figure-of-eight coil (70 mm diameter) connected to a monophasic Magstim stimulator (Magstim Company Ltd., London, UK). The settings were configured to the resting motor threshold (RMT) multiplied by 80%. Intermittent Theta Burst Stimulation (iTBS), a specific form of TMS, consists of three pulses at a frequency of 50HZ, repeated every 200 ms. Each iTBS session includes a short burst lasting 2 s followed by an 8-s break, totaling 300 pulses per session. A total of 1800 pulses were provided, with three iTBS applied to both the left and right cerebellar locations, each spaced five minutes apart. Every patient received treatment at the same time each day. The stimulation coil was affixed to the robotic arm at a 45°angle inclination relative to the midsagittal surface, with the center of the coil oriented to activate both sides of the cerebellum. The control group received sham stimulation. Treatment was conducted over 10 days, spanning two consecutive weeks, with rest on Saturdays and Sundays, once daily.

### Clinical assessments

The four scales were assessed at four time points: prior to the experiment (T0), the day following the experiment (T1), 2 weeks after T1 (T2), and 4 weeks after T1 (T3). In order to prevent potential bias, each patient underwent stimulation at various times throughout the day, ensuring unbiased data. The primary outcome measure was the scale for the assessment and rating of ataxia (SARA), which was used to assess the severity of ataxia and motor function. The secondary outcomes included the Fatigue Severity Scale-9 (FSS-9), the Hamilton Depression Scale-24 (HAMD-24), and the Hamilton Anxiety Scale (HAMA), which were utilized to assess the severity of fatigue, depression and anxiety.

### Side effects

The adverse effects commonly associated with the use of TMS devices can be categorized as follows: symptoms such as headache, pain, nausea, dizziness, and other unspecified symptoms. The trial should be terminated upon the occurrence of certain side effects.

### Statistical analysis

The experimental data were analyzed by SPSS version 22.0 (IBM, Chicago, IL, USA). The demographic data were shown as the mean ± SD for continuous variables, and as the ratio for categorical variables. The outcome indicators were presented as the mean ± SD in the tables. For within-group comparisons of normally distributed scale scores, a paired t-test was employed. To compare scores between two different groups, a two-sample independent t-test was utilized. For non-normally distributed scale scores, the rank sum test was applied. A two-way repeated ANOVA analysis was conducted with rTMS/Sham group as the between-subjects factor and time (T0, T1, T2, T3) as the within-subjects factor to evaluate the effect of rTMS on clinical outcomes. A significance threshold of α = 0.05 was used to determine statistical significance. P-values < 0.05 was indicated a statistically significant difference.

## Results

### Participants

Among eligible screened patients with potential MSA-C, 26 patients (13 in each group) completed a 10-day intervention and 4-week follow-up for scale assessment. Table 1 presents a statistical analysis of the pre-treatment demographic features of both the study group and the control group. The experimental group and the control group exhibited comparable baseline characteristics, encompassing age, sex, length of disease, MMSE, MoCA, GDS, ESS, ADL, SARA, FSS, HAMA, and HAMD. There was no statistically significant difference in the level of ataxia between the two groups (*p* > 0.05).

**Table 1:** Demographic and clinical features of participants

Variables	rTMS group (n = 13)	Sham group (n = 13)	*P*
Gender (female/male)	6/7	6/7	1.000
Age	66.92 ± 3.86	66.69 ± 4.29	0.887
Duration of illness	6.46 ± 0.97	6.08 ± 0.86	0.295
MMSE	29.08 ± 1.19	29.15 ± 0.80	0.848
MoCA	29.08 ± 0.76	29.08 ± 0.76	1.000
GDS	17.46 ± 3.84	16.85 ± 3.65	0.679
ESS	11.92 ± 2.56	13.23 ± 2.98	0.242
ADL	35.62 ± 8.87	33.85 ± 7.76	0.593
SARA	19.23 ± 4.48	19.42 ± 4.44	0.913
FSS-9	39.15 ± 2.48	38.77 ± 1.74	0.651
HAMA	23.62 ± 5.82	24.23 ± 6.04	0.794
HAMD-24	22.54 ± 7.66	23.23 ± 8.53	0.829

**Notes**: Continuous variables are represented by Means and standard deviations. **Abbreviations**: *MMSE*, Mini-Mental State Examination; *MoCA*, Montreal Cognitive Assessment; *GDS*, Geriatric Depression Scale; *ESS*, Epworth Sleepiness Scales; *ADL*, activities of daily living; *SARA*, Scale for the Assessment and Rating of Ataxia; *FSS-9*, Fatigue Severity Scale-9; *HAMA*, Hamilton Anxiety Scale; *HAMD-24*, Hamilton Depression Scale-24; *MSA-C*, Cerebellar subtype of Multiple system atrophy; *MSA-P*, Parkinsonism subtype of Multiple system atrophy

### Clinical efficacy: primary outcomes

**Fig. 2:**
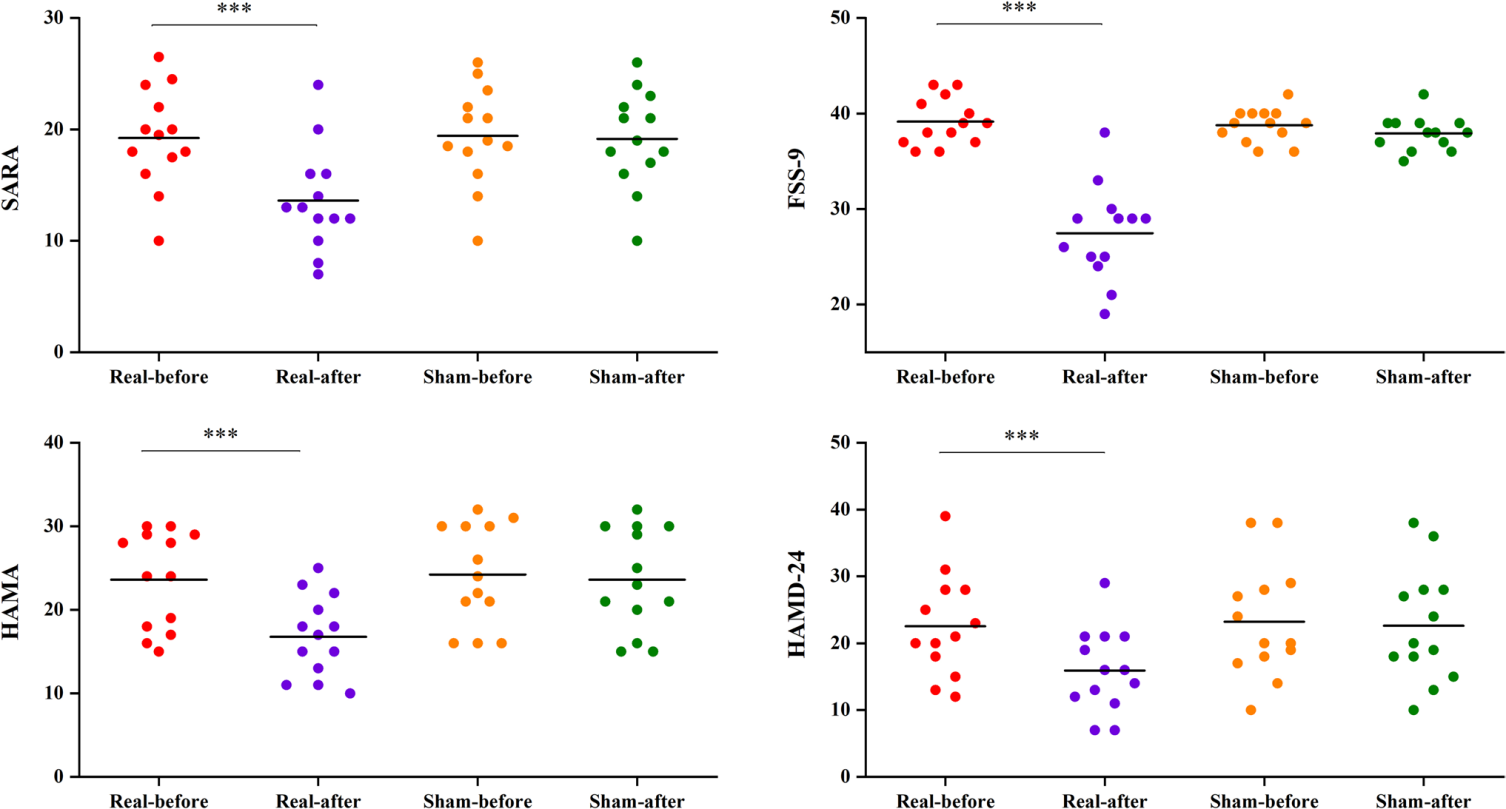
The scale score changes at different time nodes in SARA, FSS-9, HAMA, HAMD-24

The SARA score showed substantial Group × Time interactions (*p* < 0.001) and significant main effects of time (*p* < 0.001). The post-hoc analysis conducted that the experimental group exhibited substantial improvement in SARA scores at T1, T2, and T3 compared to the initial measurement at T0 (Table 2). As illustrated in Fig. 2, the score of the experimental group (T0 VS T1, 19.23 ± 4.48 vs 13.62 ± 4.63) was significantly lower than that of the control group (T0 vsT1, 19.42 ± 4.44 vs 19.15 ± 4.36). The difference between the two groups was statistically significant *(p* < 0.01).

**Table 2:** Clinical efficiency of the rTMS and Sham group

	**rTMS group (n = 13)**	**Sham group (n = 13)**		***DF***	***F***	***P***
SARA						
T0	19.23 ± 4.48	19.42 ± 4.44	Group	1	3.291	0.082
T1	13.62 ± 4.63	19.15 ± 4.36	Time	2.165	114.239	< 0.001
T2	15.38 ± 4.48	19.77 ± 4.46	Group × Time	2.165	78.972	< 0.001
T3	17.92 ± 4.46	20.46 ± 4.56				
FSS-9						
T0	39.15 ± 2.48	38.77 ± 1.74	Group	1	17.111	< 0.001
T1	27.46 ± 4.98	37.92 ± 1.80	Time	1.809	64.322	< 0.001
T2	33.69 ± 3.71	38.85 ± 1.63	Group × Time	1.809	46.732	< 0.001
T3	38.15 ± 2.85	39.00 ± 2.74				
HAMA						
T0	23.62 ± 5.82	24.23 ± 6.04	Group	1	3.723	0.066
T1	16.77 ± 4.83	23.62 ± 6.17	Time	1.400	81.065	< 0.001
T2	18.46 ± 5.21	24.31 ± 6.13	Group × Time	1.400	53.300	< 0.001
T3	21.15 ± 5.73	25.15 ± 6.14				
HAMD-24						
T0	22.54 ± 7.66	23.23 ± 8.53	Group	1	1.850	0.186
T1	15.92 ± 6.25	22.62 ± 8.50	Time	1.369	68.541	< 0.001
T2	18.08 ± 6.60	23.46 ± 8.32	Group × Time	1.369	40.059	< 0.001
T3	20.77 ± 7.44	24.46 ± 8.47				

**Notes**: Continuous variables are represented by Means and standard deviations. **Abbreviations:** *SARA*, Scale for the Assessment and Rating of Ataxia; *FSS-9*, Fatigue Severity Scale-9; *HAMA*, Hamilton Anxiety Scale; *HAMD-24*, Hamilton Depression Scale-24

### Clinical efficacy: secondary outcomes

**Fig. 3:**
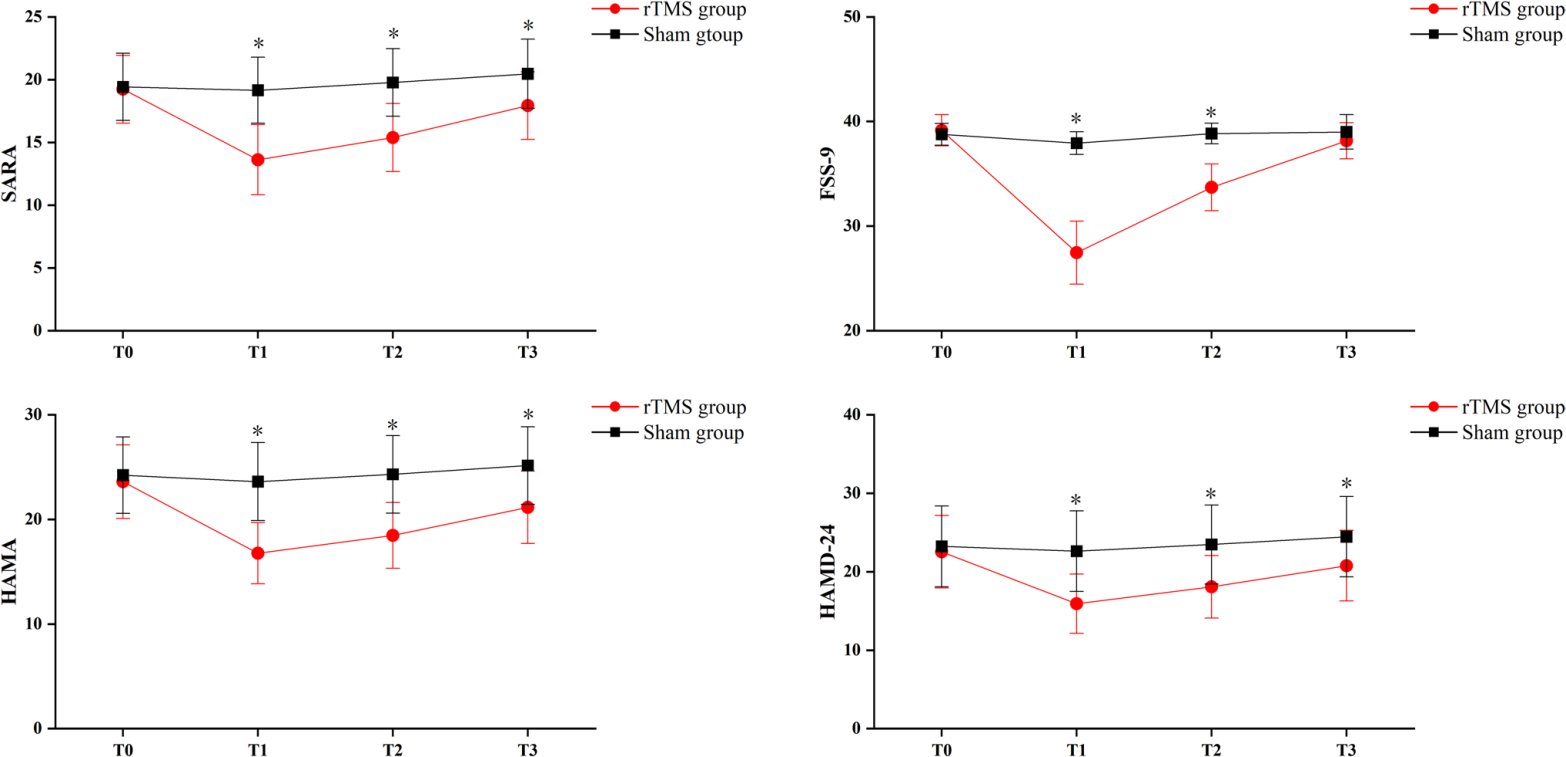
Differences in SARA, HSS-9, HAMA, HAMD-24 scores of the pre- and post-treatment in the real rTMS and sham group separately

As shown in Table 2 and Fig. 3, significant Group × Time interactions were found in FSS-9 (*p* < 0.001), HAMA (*p* < 0.001), and HAMD-24 (*p* < 0.001), indicated that the experimental group showed significant improvements in these scores compared to the sham-rTMS group. There were significant group (*p* < 0.001) and time (*p* < 0.001) main effects on FSS-9, but only time main effects on HAMA and HAMD-24 (*p* < 0.001). Post-hoc analysis after treatment showed that compared with T0, the scores of T1, T2 and T3 in the experimental group were significantly reduced, with a greater degree of reduction compared to the control group at T1 (Fig. 3).

## Discussion

In this study, rTMS was applied to the bilateral cerebellum of MSA-C patients. The main findings of this study are as follows: (1) rTMS substantially reduced the SARA scores at T1, T2, and T3 compared with the initial measurement at T0. (2) In real rTMS, the changes in scale scores were most significant at T1 and gradually diminished with longer follow-up. This change was revealed to have a significant temporal correlation. The SARA score substantially decreased at T1, T2 and T3, indicating a notable improvement in symptoms of ataxia among the patients, which is consistent with Song's findings[6]. Their clinical trial in which rTMS was applied over the cerebellum of patients with MSA revealed that the improvement in ataxia symptoms with real rTMS was more obvious. Recently, G. Koch et al.[7] reported that patients suffering from hemiplegia following a stroke experienced improved gait and balance after undergoing three weeks of iTBS targeting the cerebellum. In a meta-analysis on hereditary ataxia, five RCTs applying the SARA scores to assess ataxia were included. The results all suggested a significant reduction in SARA scores from baseline to postintervention when TMS was applied to the cerebellum compared with sham rTMS[8].

Furthermore, our research revealed that stimulating the cerebellum alleviated anxiety, despair, and exhaustion among patients diagnosed with MSA-C. In this study, the focus was on the cerebellum, which has been found to play an important role in regulating exercise, fatigue and cognition in recent years[9]. Therefore, we hypothesized that the cerebellum may also affect anxiety and depression in patients with MSA-C. Pan reported that high-frequency rTMS could alleviate fatigue in patients with MSA[4]. A study of patients with multiple sclerosis and cerebellar atrophy revealed that rTMS applied to the cerebellum can effectively alleviate early fatigue and depression symptoms, which is also consistent with the results observed in our trial[10].

In general, ataxia is the most prominent and common symptom in patients with MSA-C, and fatigue and emotional problems often accompany this ataxia[11]. The area of focus of this study was the bilateral cerebellum, which is an important brain region that regulates movement and body balance[12]. The cerebellum plays a key role in human fatigue[13] and emotional regulation[14–17]. In recent decades, extensive research has consistently demonstrated the involvement of the cerebellum in regulating emotional and cognitive engagement. The researchers reported greater improvements in clinical symptoms and HAMD scores in the experimental group than in the control group, with an interaction effect between group and time (before and after treatment), which is also consistent with the results of our study[18]. This finding is also in agreement with that of Katharina M., who reported that rTMS stimulation of the cerebellum reduced the emotional problems of patients with major depression[19]. Conventionally, TMS can stimulate the cerebellar cortex, activate Purkinje cells, and inhibit the M1 area to help patients control uncoordinated movements[20]. Previous studies have also shown that TMS can regulate and treat cerebellar ataxia, compensate for the insufficient inhibitory function of cerebellar nuclei caused by the loss or functional impairment of Purkinje cells, and transiently excite of cortical inhibitory interneurons in the cortical motor area, which is manifests as a prolonged motor threshold and a cortical silent period[21, 22]. These findings indicate that the activation of the cerebellum can effectively regulate the activity of the cortex or network in many manners, hence providing additional potential for the treatment of neurological disorders.

There are relatively few experiments related to MSA-C and TMS, and only the immediate effect was evaluated in most of the studies, without follow-up. We therefore sought to extend the follow-up period as long as possible to explore the durability of improvement at 1 month. The initial findings of Manor B et al.[23] are also consistent with our findings that the application of rTMS to the cerebellum produced beneficial effects on spinocerebellar ataxia patients that persisted for a minimum of 1 month. So as J.Pan et al.[4] suggest that the rTMS over left DLPFC may alleviating fatigue in MSA patients, though the beneficial effects last no more than 4 weeks. This difference may be due to differences in the parameters used in our study as well as the duration of treatment[24]. More importantly, MSA is characterized by persistent worsening of motor and nonmotor symptoms with more rapid progression at onset[25]. This suggests that at the time of follow-up, the patient may have experienced an exacerbation of both motor and nonmotor symptoms. Nevertheless, in the control group that received sham-rTMS, there was a slight decrease in scale scores. The observed outcomes could be attributed to the placebo effect in the control group[26].

However, the main limitations of this study are the small number of patients evaluated and the short follow-up period. We recommend further studies with larger sample sizes and longer follow-up periods. Nevertheless, additional research is needed to ascertain the underlying mechanism that influences the symptoms of ataxia, fatigue, anxiety, and depression in patients with MSA-C.

## Conclusions

 In conclusion, our findings provide novel evidence that rTMS applied bilaterally to the cerebellum may serve as an effective intervention for improving ataxia and alleviating fatigue, anxiety, and depression in MSA-C patients. However, the beneficial effects appear to be limited to a duration of no more than 4 weeks. Our study also proposes a novel therapeutic approach that may alleviate symptoms in patients with MSA. In the future, more positive rTMS procedures and protocols will be required to extend the treatment effect in general clinical practice.

## Data Availability

Experimental data are available upon request from the corresponding authors.
